# Jordanian Use of and Beliefs Concerning the Efficacy of Medicinal Plants: A Cross-Sectional Study

**DOI:** 10.7759/cureus.37494

**Published:** 2023-04-12

**Authors:** Kanar Sweiss, Abdallah Y Naser, Abdel-Rahman Tayseer

**Affiliations:** 1 Basic Pharmaceutical Sciences, Faculty of Pharmacy, Isra University, Amman, JOR; 2 Applied Pharmaceutical Sciences and Clinical Pharmacy, Faculty of Pharmacy, Isra University, Amman, JOR

**Keywords:** utilisation, plants, medicinal, jordan, herbs

## Abstract

*Background:* Medicinal herbs are incorrectly believed to be free of risks and are commonly used for self-medication without medical supervision. Jordan does not currently have a national policy on traditional medicine (TM) and/or complementary/alternative medicine (CAM). The present study aims to explore the use of and beliefs about the efficacy of medicinal plants among the Jordanian population.

*Method:* A cross-sectional study was conducted using a self-administrated questionnaire for the duration between April and June 2019. Multiple linear regression analysis was used to identify predictors of positive attitudes towards the use of medicinal plants.

Results: A total of 1,057 individuals participated in the study. The participants in our study showed a positive attitude towards the use of medicinal plants and herbs (median score of 33.0 (interquartile range (IQR): 26.0-37.0; equals 68.8% of the maximum total score), and believed in alternative therapies other than chemical drugs for disease treatment, mainly using medicinal herbs and plants. The majority of the participants (77.8% (n = 822)) believe in the efficacy of the use of medicinal herbs and plants and are aware (64.6 % (n = 683)) of the correct and proper way of using these plants and herbs. Pharmacists and herbalist are the main sources of information for the proper use of medicinal herbs and plants. Age was the main predictor of positive attitudes towards the use of medicinal plants and herbs (P<0.001).

*Conclusion:* Efforts must be made to regulate the dispensing of these products, to educate health service providers and to raise consumer awareness.

## Introduction

 Across time, nature was the main source of human basic needs for the production of foodstuff, shelter, transportation, clothing, flavors, fertilizers, fragrances, and medicines. For thousands of years, nature has been a source of medicinal products, and an large number of contemporary medications have been isolated from natural sources, emerging from their use in traditional medicine (TM) [1]. Therefore, traditional medicinal plants are considered as an important element of indigenous medical systems around the world [2].

Over the past three decades, there has been a significant reinforcement of herbal medicine [3]. Many medicinal herbs are believed to be risk-free and are commonly used for self-medication without any needed medical supervision. Another strength of herbal medicines is that they are cheaper than conventional treatments. The World Health Organization (WHO) estimates that 80% of developing countries population depend on traditional herbal medicines [4]. However, the "naturalness" of medicinal herbs does not mean that they are risk-free. Potential toxic factors, consumers-related factors such as age, pregnancy, disease factors, contamination, adulteration, and lack of regulation are risk factors that are considered to be associated with harmful herbal adverse reactions, poisonings, and other complications [5,6]. Moreover, interactions with conventional drugs are possible, leading to a decrease in the effectiveness of pharmacological treatments or to toxic manifestations [7]. In 2002, the WHO has declared that efficacy, and safety of traditional and complementary/alternative medicine (CAM) have become important concerns for both health authorities and the general public. Only 25 among the WHO’s 191 member states have developed policies that regulate the utilization of TM and CAM. Therefore, a number of issues must first be tackled to maximize the potential of their use in health care [8]. In a global report by the WHO that was released in 2019, Jordan was reported as not currently having a national policy on TM/CAM; however, laws and regulations which had been established in 2001 were updated in 2016 [9]. In addition, there are no independent national research institutes that have been established on TM/CAM or herbal medicines. The regulations of herbal products are partly the same as those for conventional pharmaceuticals. Herbal products are regulated as over the counter (OTC) medicines. By law, claims can be made for medical, health, nutrient content and structure/function. Instead of the National Pharmacopoeia, the United States Pharmacopoeia is used. The WHO monographs are used in place of national monographs, and are considered legally binding [9].

In Jordan, the requirements that regulate the manufacturing of herbal medicines follow the same rules of Good Manufacturing Practice (GMP) that apply to conventional pharmaceuticals; execution is ensured by through quality control. The requirement of safety assessment are the same as for conventional pharmaceuticals, but they also include special requirements: conventional use must not show any adverse effects and reference should be made to documented scientific research on similar products. A control mechanism exists for these requirements, involving toxicity studies. The register of herbal medicines contains 76 medicines. There are no herbal medicines included in the national essential medicines list. In Jordan, herbal medicines are sold in pharmacies as OTC and non-prescription medicines without restriction [9]. The present study aims to explore the use of and beliefs about the efficacy of medicinal plants and herbs among the Jordanian population.

## Materials and methods

Study design

A cross-sectional study was conducted using a self-administered questionnaire for the duration between April and June 2019. The study was conducted in different governorates in Jordan, representing both low and high socioeconomic areas.

Data collection

The questionnaire was developed by the principal researcher after an extensive literature review in the area of the use of herbal medicine. The external validity of the questionnaire was checked by research experts and academics in the area of clinical pharmacy and pharmacy practice. It was then piloted with 15 pharmacy students to test for the clarity of the questions. The participants in the pilot study confirmed that it is straightforward and easy to understand. No change was made on the questionnaire tool.

The questionnaire was administered to the eligible participants by pharmacy students in their final year using convenience sampling technique. Students were selected on voluntary basis. Any student who was interested in distributing the questionnaire was involved. The eligible participants were individuals aged 18 years and above, who got their medication from different resources such as community pharmacies, hospital pharmacies in the military, public and private hospitals. The students who helped in collecting the data were trained on how to approach the participants to ensure uniformity and accuracy in data collection. The importance of the originality of the data was explained to them before the study commenced.

The questionnaire tool

The questionnaire was composed of three parts. The first part addressed the participants’ demographics by asking about gender, age, educational level, their residential location, marital status, employment status, their income, and if the participant suffered from any diseases. The second part of the questionnaire included eight "Yes or No" format questions and one open-ended question regarding the medicinal plant most commonly used by the participants. The third part of the questionnaire was composed of 14 questions. Twelve questions (using a 5-point Likert scale) included in this part (the part that measured attitudes to and beliefs about the efficacy of medicinal plants and herbs) were prepared to draw specific information from the participants regarding many issues, including if they were they happy to have medicinal herbs in their homes, if medicinal herbs were their first choice for treatment, if they had greater trust in any medication extracted from medicinal herbs, if they liked to have both natural and chemical treatment in parallel, along with other questions related to their use, beliefs, and knowledge concerning the use of herbal medicine. The participants’ responses ranged from 0 to 4, where 0 meant “strongly disagree” and 4 meant “strongly agree”. The total possible score for this part of the questionnaire could range between 0 and 48 and could be interpreted based on the mid-point of the highest possible score of the scale (equal to 24). The higher the score, the more positive the attitudes and beliefs regarding the use of medicinal plants and herbs were. In addition, the participants were asked whether they believed that “medicinal plants and herbs are not a substitute for treatment but rather an aid to it” and whether they were “satisfied with the herbalist's advice as it is or they would rather search for the correct way to use medicinal plants and herbs through the internet”.

Sample size

The website Survey Monkey was used to determine the sample size necessary for this research. Based on a confidence interval of 95%, a SD of 0.5, an error margin of 5%, and a population of more than 10 million, the minimum required sample size was 385 individuals.

Ethical approval

Ethical approval for this study was obtained from the research ethics committees (REC) at the Faculty of Pharmacy at Isra University in Jordan (PH - 2019 - 13).

Statistical analysis

Data were analyzed using SPSS software, version 27. Continuous data was reported as mean (SD) for normally distributed quantitative and as median + interquartile range (IQR) for non-normally distributed quantitative variables. Descriptive statistics were used to describe the participants’ demographic information. Categorical data were reported as percentages and frequencies. The median score (IQR) for each item was calculated based on the participants’ response using a five-point Likert scale, ranging between 0 and 4. The participants’ scores were interpreted as a continuous scale based on the scale midpoint, where scores above the midpoint identified positive attitudes and beliefs concerning the use of medicinal plants. Collinearity diagnostics variance inflation factor (VIF) value for the study variables ranged between 1.04 to 1.43, which reflects that absence of strong multicollinearity between a given predictor variable and other predictor variables in the model. We have now added these details to the statistical analysis section. Multiple linear regression analysis was used to identify the predictors of positive attitudes towards the use of medicinal plants. Statistical significance was considered if the p < 0.05 with a 95% confidence interval. The internal consistency of the awareness items was measured using Cronbach's alpha, which reflected good internal consistency (α = 0.898).

## Results

Participants’ characteristics

A total of 1,057 participants responded to the questionnaire; the mean age of the participants was 33.4 (SD = 13.4) years, ranged between 18 and 90 years. The majority of the participants (77.5%, n = 819) were located in the capital Amman (the capital) and 61.6% (n = 651) held a bachelor’s degree. Around half of the participants (50.7% (n = 536)) were single and employed (52.8% (n = 558)). The majority of the participants (61.6% (n = 651)) reported that their income was below 500 Jordanian dinar (JD) per month. When the participants answered the question related to their medical records and any diseases they were suffering from, 17.7% (n = 187) reported that they were suffering from high blood pressure, 15.4% (n = 163) were diabetic patients, and 11.5% (n = 122) had high levels of lipids and cholesterol (Table 1).

**Table 1:** Characteristics of the study participants JD: Jordanian dinar

Demographic variable	Percentage	Frequency
Age (Mean (SD)) years		
33.4 (SD = 13.4)		
Educational level		
Secondary school or lower	30.9	327
Diploma	2.5	26
Bachelor degree	61.6	651
Higher education	5.0	53
Marital status		
Single	50.7	536
Married	41.8	442
Divorced	4.4	46
Widow	3.1	33
Employment status		
Employed	52.8	558
Unemployed	47.2	499
Monthly income		
Below 500 JD	61.6	651
500-1,000 JD	27.1	286
1,000-1,500 JD	7.6	80
More than 1,500 JD	3.8	40
Comorbidities		
Hypertension	17.7	187
Diabetes mellitus	15.4	163
Dyslipidemia	11.5	122
Arthritis	9.7	103
Gastric ulcer	8.8	93
Cancer	0.4	4

Beliefs about medicinal herbs and plants

Around 74.6% (n = 789) of the participants reported that they believed in alternative therapies other than chemical drugs for disease treatment, 64.6% (n = 683) believed in using medicinal herbs and plants, while 13.0% (n = 137) believed in acupuncture and 10.1% (n = 107) believed in religious means of curing diseases. More than half of the participants (57.2 (n = 604)) reported that they had previously used medicinal herbs and plants for the treatment of a range of diseases, including abdominal pain, headache, flu, diarrhea, cough, diabetes, arthritis, blood pressure, bronchitis, and constipation (Table 2).

**Table 2:** The most common disease to be treated by the use of medicinal herbs and plants by the participants

Disease	Percentage	Frequency
Abdominal pain	16.3	172
Flu	10.7	113
Diarrhea	3.7	39
Cough	3.3	35
Diabetes	3.3	39
Arthritis	2.2	23
Headache	2.1	22
Blood pressure	1.8	19
Bronchitis	1.7	18
Constipation	1.6	17

A majority of the participants (77.8% (n = 822)) believed in the efficacy of the use of medicinal herbs and plants, and 64.6 % (n = 683) claimed that they knew the correct and proper way of using these herbs. Around 22.6% (n = 235) of the participants reported that the pharmacist was their main source of information for the proper use of these medicinal herbs and plants, and 22.5% (n = 234) got directions of use from a herbalist (Table 3).

**Table 3:** Source of the correct and proper direction for the use of medicinal herbs and plants

Source of correct directions of use	Percentage	Frequency
Pharmacist	22.6	235
Herbalist	22.5	234
Web sites	19.2	200
Doctor	15.3	159
Nurse	3.9	40

Utilization of medicinal herbs and plants

Only 25.7% (n = 267) of the participants who used medicinal herbs and plants as treatment for certain diseases reported that they had suffered from side effects such as abdominal pain 11.2% (n = 118), diarrhea 10.8 (n = 113), and vomiting 7.1% (n = 75). Around 58.8% (n = 622) of the participants were interested in being educated and in reading more about new information concerning the use of medicinal herbs and plants in treating diseases instead of using chemical drugs. When searching for such information, 38.3% (n = 396) depended on Facebook, 27.1% (n = 289) depended on the Google search engine, while 13.2% (n = 136) used Instagram. The participants used medicinal herbs and plants to overcome some common health problems, especially abdominal pain, flu, diarrhea, gastric ulcers and arthritis. Of those, only 47.0% (n = 497) bought these herbs and plants from special shops (Table 4). Around 68.0% (n= 719) of the participants reported that they believed that medicinal plants and herbs were not a substitute for treatment but rather an aid to it. Besides this, 70.5% (n= 745) of the participants reported that they were not satisfied with the herbalist's advice (quality of advice) about the use of medicinal plants and herbs and that they would rather search for the correct way to administer them through the internet.

 

**Table 4:** The most common health problems that the participants use medicinal herbs and plants to overcome

The common health problems the participants overcome by medicinal herbs and plants	Percentage	Frequency
Abdominal pain	42.2	445
Flu	38	401
Diarrhea	27.8	293
Gastric ulcer	13.8	146
Arthritis	11.2	118
Blood pressure	6.3	66
Diabetes	6	63
High levels of lipids	3.3	35

Sage, mint, and chamomile were the most commonly reported plant to be used by the study participants. Table 5 presents the most commonly used medicinal herbs and plants by the study participants.

**Table 5:** The most commonly used medicinal herbs and plants by the participants

The most commonly used medicinal herbs and plants	Percentage	Frequency
Sage	25.6	271
Mint	16.2	171
Chamomile	15.8	167
Anise	11.2	118
Thymus	3.8	40
Cinnamon	3.7	39
Germander	3.2	34
Lavender	3.0	32
Lemon	2.6	28
Curcuma	2.1	22
Garlic	1.4	15
Green tea	1.1	12

Predictors of positive attitude towards the use of medicinal plants 

The total scores of the participants’ questionnaires ranged from zero to 48, with a median score of 33.0 (IQR = 26.0 - 37.0) (equal to 68.8% of the maximum total score). As the median score of the participants was above the midpoint of the highest possible score of the questionnaire (which is 48), this shows that they have positive attitudes and beliefs regarding the use of medicinal plants and herbs. A simple linear regression showed that older age, being married or widowed, and being employed were significantly associated with positive attitudes towards the use of medicinal plants (P<0.01). On the other hand, a high income (1,500 JD and above) and being single were negatively associated with participant attitudes towards the use of medicinal plants (p<0.01). Multiple regression analysis was conducted using two models using the participants’ demographic characteristics that we expected to influence their attitude towards the use of medicinal plants; the first one included participants age, gender, and education, and the second included the participants’ age, gender, and residential location. A confidence interval of 95% (P<0.05) was applied to represent the statistical significance of the result and the level of the significance was assigned as 5%. The two models confirmed that age was a predictor of positive attitudes and beliefs regarding the use of medicinal plants and herbs (P<0.001).

## Discussion

This was a comprehensive study seeking to reflect the utilization pattern and beliefs concerning medicinal plants and herbs among participants from different age groups and socioeconomic and educational levels. The key findings are: 1) the participants in our study showed a positive attitude towards the use of medicinal plants and herbs (median score of 33.0 (IQR: 26.0-37.0; equals 68.8% of the maximum total score), and believed in alternative therapies other than chemical drugs for disease treatment, mainly using medicinal herbs and plants; 2) a majority of the participants believe in the efficacy of the use of medicinal herbs and plants, and are aware of the correct way of using these plants and herbs; 3) pharmacists and herbalist are the main source of information for the proper use of medicinal herbs and plants; 4) around 25.7% (n = 267) of the participants who used medicinal herbs and plants as a treatment for certain diseases reported that they had suffered from side effects; and 5) age was the main predictor of a positive attitude towards the use of medicinal plants and herbs.

Confirming the findings of previous studies conducted in Jordan, our study revealed a positive attitude to and belief in the use of medicinal plant and herbs, which has resulted in widespread use of various types of these natural products for the treatment of various health conditions [10-12]. Using medicinal plants and herbs without structured guidelines and regulations that clarify and guide the proper use of these products is associated with various alarming consequences [13]. Several reports have highlighted the risk profile associated with the use of herbal products based on laboratory test findings. Effects such as complications in the management of other comorbidities the patient may be suffering from, direct toxicity (including cardiac, pulmonary, renal, hematological, hepatic and endocrine), in addition to potential drug-herb interactions [13-17]. There are multiple drug-herb interactions that are associated with the use of herbal products among patients suffering from other comorbidities and which are associated with a wide range of serious side effects [18-21]. The above-mentioned risk profile accompanied by the low level of education among herbalists that has been reported in multiple studies will ultimately increase the complexity of the use of herbal products and the associated risks [10,22,23]. Our study confirms the findings of a previous study in Jordan by Issa and Basheti that stated herbalists and pharmacists were the main sources of information concerning the use of herbal products [10]. However, our study population gave the same weight to pharmacists as the main source of information, contradicting Issa and Basheti’s study, which reported priority to herbalists as the main source of information over pharmacists [10]. Our study emphasizes the fact that pharmacists are increasingly becoming the trustworthy source of information for patients, whether for OTC medications, prescription medications or herbal products, as they are expected to have access to comprehensive science-based data about all kinds of treatments, whether chemical or natural.

Our study population reported the use of medicinal plants and herbs for various health conditions (both acute and chronic). The main reported conditions were abdominal pain, flu, and diarrhea. Surprisingly, there is a proportion of the Jordanian population who use medicinal plants and herbs for the treatment of chronic conditions, such as diabetes mellitus, hypertension and dyslipidemia. A previous study in Jordan confirmed our study findings when reporting the common belief of the Jordanian population in the efficacy of medicinal plants and herbs in treating a wide variety of chronic conditions [11]. Although the majority of the reported plants and herbs used among our study population were safe, none of them proved its efficiency in treating chronic diseases such as diabetes mellitus, hypertension, or dyslipidemia, or to be a substitute for the currently administrated pharmacological therapies [11]. It was previously reported that there is confusion about the identification, therapeutic dosage, effectiveness, toxicity, standardization, and regulation related to the use of medicinal plants and herbs [24].

In our study, around 25.7% (n = 267) of the participants who used medicinal herbs and plants as treatment for certain diseases reported that they had suffered from side effects, which mainly affected the gastrointestinal tract in the form of abdominal pain, diarrhea and vomiting. This was in line with a previous study in Jordan; however, our study showed a higher prevalence of side effects (25.7%) compared to theirs (19.9%) [10]. Healthcare providers and, specifically, pharmacists should better educate patients on the proper use of medicinal plants and herbs as they are the most commonly reported source of information related to the use of these products among the general population. To facilitate the pharmacists’ role in delivering the safer use of medicinal plants and herbs, they should possess enough and accurate information on their appropriate use. This can be achieved through providing continuous medical education courses for pharmacists who have already graduated and currently practicing their profession, and by increasing the focus of the pharmacy curriculum on the use of medicinal plants and herbs for the treatment of different health condition for pharmacy students. Such practices should promote the safer use of these natural products.

Our study has revealed that more than half of the study population (around 58.8% (n = 622)) were interested in being educated and in learning more about the safety and effectiveness of medicinal herbs and plants. This positive attitude towards learning more is reflected in the desire to launch awareness campaigns involving cooperation between pharmacists, herbalists and doctors, and publishing brochures that can be distributed in herbal shops, pharmacies and clinics or on formal medical websites to elaborate the right, safe and effective way of using these herbal products under the supervision of the Ministry of Health.

Our study showed that age was the only significant predictor of having a positive attitude to and belief in the use of medicinal plants, which implies that the positive attitude and belief in the effectiveness of the medicinal plants increases with age. This positive attitude and belief could be due to the experience of using herbal products in treating different minor illnesses that the elderly are prone to. The elderly population tends to have a positive attitude towards the use of herbal products due to their health profile, which is characterized by having multiple comorbidities. Having multiple comorbidities and the associated polypharmacy forces them to search for alternative (non-chemical) remedies, which explains the remedies’ popularity.

This study has several strengths. This is the first study in a Middle Eastern Arabic-speaking country to explore the predictors of positive attitudes towards the use of medicinal plants and herbs. The study population included participants using medicinal plants and herbs for any indication, without any restrictions as to specific disease populations or specific governorates. This should increase the generalizability of these findings. The large sample size of our study population increases the accuracy of our estimates and decreases the margin of error. However, this study has some limitations. The cross-sectional study design itself limited our ability to detect causality between study variables. We were not able to estimate the response rate for our questionnaire study as we did not estimate the number of participants who were invited to participate in our study, which might lead to non-response bias as we could not demonstrate how well the sample had been drawn from the population of interest. This study relied on self-reported data from participants, which may be subject to recall bias, social desirability bias, or inaccuracies. The majority of the participants (77.5%) were located in the capital city, Amman. This might limit the generalizability of the study findings, as people living in other regions or rural areas may have different attitudes, beliefs, or access to medicinal herbs and plants. Therefore, the findings should be interpreted carefully.

## Conclusions

Despite the lack of clear and structured guidelines and regulations that clarify the proper use of these herbal products in Jordan, their use is very common among the Jordanian population. Our findings showed that a significant proportion of participants relied on social media platforms (Facebook, Instagram) and search engines (Google) for information on medicinal herbs and plants. This raises concerns about the quality and accuracy of the information they may be exposed to, as these sources can contain misinformation. Efforts have to be made to regulate the dispensing of these products, to educate health service providers and to raise consumer awareness. This could be overseen by the Ministry of Health, the medical bodies, and universities, resulting in increased awareness about the safe and effective way to use these medicinal products among all actors: herbalists, doctors, pharmacists, and, most importantly, patients.
